# Pathological complete response after monotherapy with immune checkpoint inhibitors for bifocal colon cancer in a patient with lynch syndrome and situs inversus totalis: a case report

**DOI:** 10.3389/fimmu.2025.1571607

**Published:** 2025-04-22

**Authors:** Jun Feng, Wenbo Niu, Juan Zhang, Yuanyi Ding, Zheng Li, Jianfeng Zhang, Baokun Li, Chenhui Li, Feifei Wang, Guiying Wang, Bin Yu

**Affiliations:** ^1^ The Second Department of General Surgery, The Fourth Hospital of Hebei Medical University, Shijiazhuang, Hebei, China; ^2^ Hebei Key Laboratory of Etiology Tracing and Individualized Diagnosis and Treatment for Digestive System Carcinoma, The Second Hospital of Hebei Medical University, Shijiazhuang, Hebei, China; ^3^ Departments of General Surgery, The Second Hospital of Hebei Medical University, Shijiazhuang, Hebei, China

**Keywords:** situs inversus totalis, lynch syndrome, immune checkpoint inhibitors, pathological complete response, colon cancer, microsatellite instability-high, immune-related adverse events

## Abstract

**Background:**

Lynch syndrome is the most common hereditary colorectal cancer (CRC) syndrome, accounting for 3–5% of all CRC cases. Situs inversus totalis (SIT) is a rare congenital malformation with an incidence of 1 in 8,000 to 1 in 25,000. The co-occurrence of Lynch syndrome and SIT is extremely uncommon. Immune checkpoint inhibitors (ICIs) have demonstrated significant efficacy in treating microsatellite instability-high/deficient mismatch repair (MSI-H/dMMR) CRC. Tumors associated with Lynch syndrome frequently exhibit MSI-H, providing a theoretical basis for ICI use.

**Case presentation:**

We report a case of bifocal colon cancer associated with Lynch syndrome and SIT. After seven cycles of sintilimab, the patient developed gastrointestinal perforation due to tumor regression, necessitating emergency surgery. The anatomical variations associated with SIT required the surgical team to adopt an alternative approach. Postoperatively, the patient continued sintilimab treatment for 2 years. In June 2024, he underwent a colostomy reversal and proximal colectomy. Pathological examination revealed a tumor regression grade (TRG) of 0, indicating complete pathological remission (pCR), with no recurrence or metastasis detected upon follow-up.

**Conclusions:**

The anatomical variations associated with SIT increase the complexity of surgical procedures. Advanced imaging modalities such as computed tomography (CT) and magnetic resonance imaging (MRI) are essential for assessing fine anatomical details and facilitating surgery. ICIs are an effective treatment option for Lynch syndrome-associated CRC, as demonstrated in this case. Future studies should investigate the optimal timing of immunotherapy, combination treatment strategies, and methods to mitigate immune-related toxicities. Such research will help develop comprehensive and personalized treatment plans for Lynch syndrome-associated CRC.

## 1 Introduction

Colorectal cancer (CRC) has the third highest incidence and is the second leading cause of cancer-related deaths worldwide (1). Lynch syndrome, an autosomal dominant hereditary disorder, is primarily caused by mutations in mismatch repair genes and accounts for 3–5% of all CRC cases (2). Patients with Lynch syndrome have an elevated risk of developing CRC and other cancers, such as endometrial and gastric cancer (3). The hereditary nature of this condition implies that family members of affected individuals may also be at risk.

Situs inversus totalis (SIT) is a rare congenital condition characterized by a mirror-image arrangement of internal organs (4). While SIT itself does not cause health issues, it can be associated with congenital anomalies or dysfunctions, such as congenital heart disease (5) and primary ciliary dyskinesia (6). The co-occurrence of Lynch syndrome and SIT is exceedingly rare, creating a unique clinical scenario.

Immune checkpoint inhibitors (ICIs) have revolutionized cancer therapy. By blocking inhibitory signaling pathways between T lymphocytes and antigen-presenting cells, ICIs activate tumor-specific T cells, enhance immune responses, improve the tumor microenvironment, restore the normal antitumor immune response, thereby achieving antitumor effects (7). Recent studies have shown that ICIs are effective in treating microsatellite instability-high/deficient mismatch repair (MSI-H/dMMR) metastatic CRC, making them a promising option for Lynch syndrome-associated CRC (8, 9).

Here, we report a rare case of bifocal colon cancer associated with Lynch syndrome and SIT. The patient achieved a pathological complete response (pCR) after ICI monotherapy, demonstrating the efficacy of ICIs in Lynch syndrome-associated CRC. However, the treatment posed several challenges, including anatomical variations due to SIT and gastrointestinal perforation caused by rapid tumor regression. Exploring ICI use in patients with CRC and Lynch syndrome not only enhances prognosis for this specific population but also provides new insights into personalized treatment approaches.

## 2 Case report

**Figure 1:**
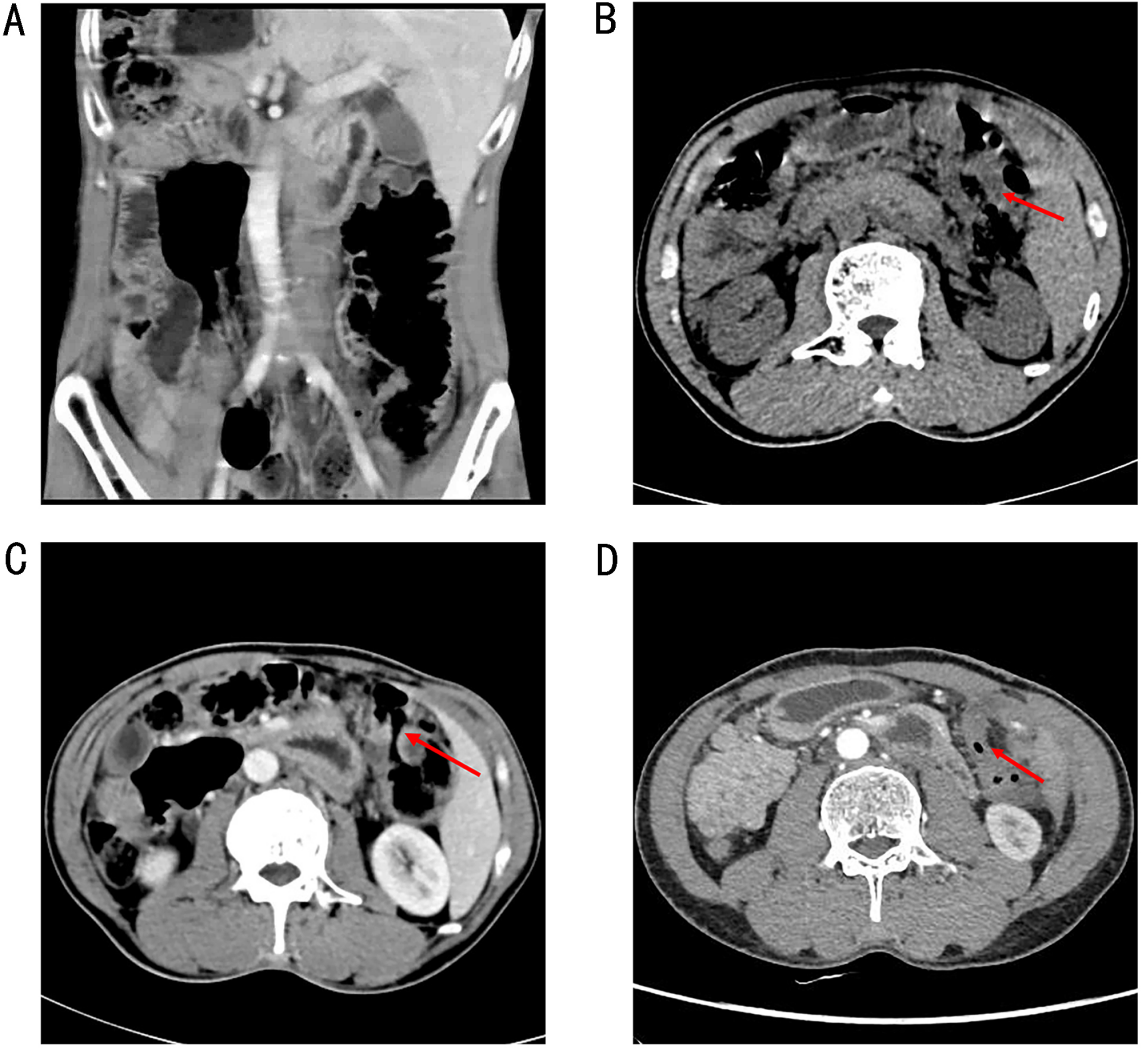
A chest and abdominal CT scan revealed SIT **(A)** and intestinal wall thickening in the hepatic flexure of the colon **(B)**. CT image showed that the thickening of the colonic hepatic flexure wall was better than before **(C)** (on April 28, 2021). CT image showed that no abnormal enhancement of the colonic hepatic flexure wall **(D)** (In September 2021).

A 48-year-old male presented to our hospital on January 26, 2021, with intermittent distension in the left upper abdomen. Chest and abdominal computed tomography (CT) revealed SIT and intestinal wall thickening at the hepatic flexure of the colon (clinical T4N1M0) (**Figures 1A, B**). Electronic colonoscopy identified neoplasms in both the ascending and sigmoid colons, and biopsies confirmed adenocarcinoma. The patient was diagnosed with bifocal colon cancer. His medical history was unremarkable. However, his family history included multiple cases of cancer: his father died of lung cancer, one sister and his brothers had been diagnosed with Lynch syndrome. His uncle had colon cancer and one cousin had a bone tumor. The genetic test done by the patient at the company showed KRAS wild-type, NRAS wild-type, BRAF wild-type, and MSI-H. Based on these findings, the patient was diagnosed with Lynch syndrome and SIT.

**Figure 2:**
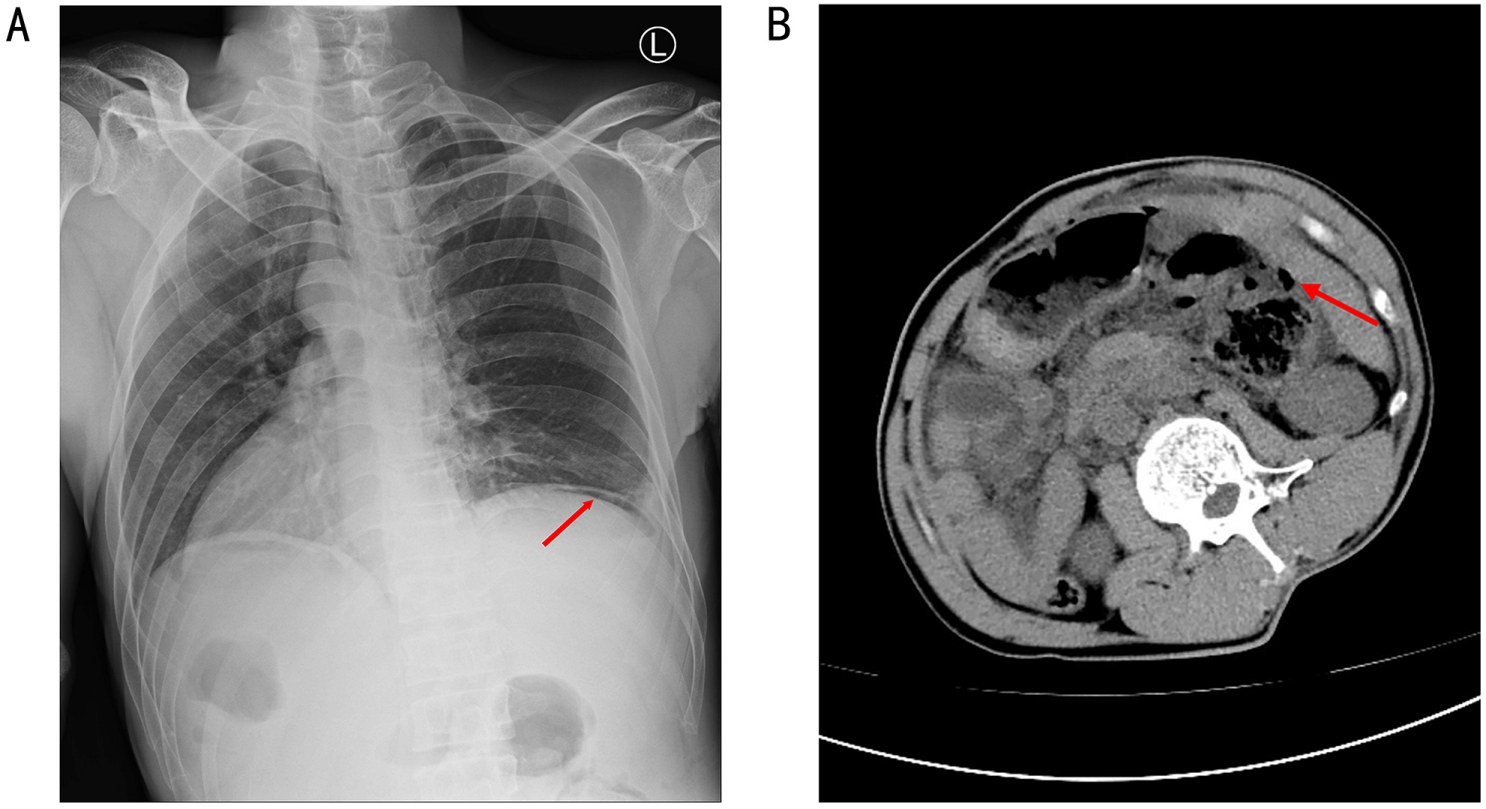
A chest and abdominal CT revealed free gas in the abdominal cavity, indicating a gastrointestinal perforation **(A, B)**.

**Figure 3:**
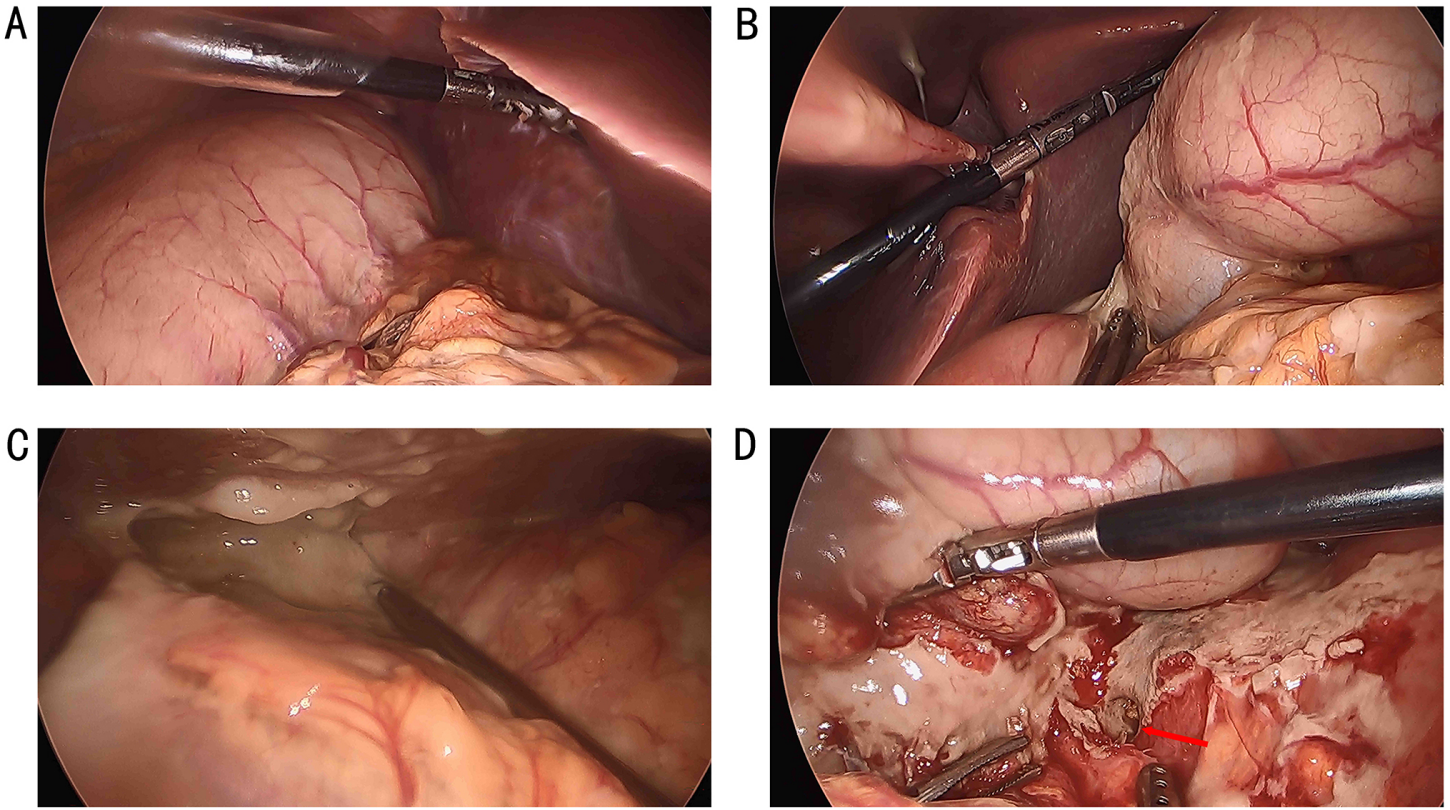
The stomach and spleen were in the right upper abdomen **(A)**, while the liver and gallbladder were in the left upper abdomen **(B)**, and the ascending and descending colon were swapped. A perforation was found in the hepatic flexure **(D)**, accompanied by a significant amount of turbid ascitic fluid in the abdominal cavity **(C)**.

The patient declined surgical treatment and opted for immunotherapy. He received intravenous sintilimab (200 mg every 3 weeks). After three cycles, a CT scan on April 28, 2021, showed improvement in the thickening of the colonic hepatic flexure wall (**Figure 1C**), and the treatment response was assessed as a partial response. However, on July 27, 2021, after seven cycles of sintilimab, the patient developed sudden abdominal pain. Chest and abdominal CT revealed free gas in the abdominal cavity, indicating gastrointestinal perforation (**Figures 2A, B**). Emergency exploratory laparotomy revealed a mirror-image arrangement of internal organs: the stomach and spleen were located in the right upper abdomen, while the liver and gallbladder were in the left upper abdomen (**Figures 3A, B**). The ascending and descending colons were also transposed. A perforation was identified at the hepatic flexure, accompanied by a significant amount of turbid ascitic fluid (**Figures 3C, D**). Due to abdominal inflammation leading to intestinal swelling, accurately assessing the tumor response intraoperatively was challenging. The patient underwent laparoscopic repair of the colonic perforation and a colostomy and was discharged on the eighth postoperative day.

In September 2021, a follow-up contrast-enhanced chest and abdominal CT scan showed no abnormal enhancement in the colonic hepatic flexure wall (**Figure 1D**). The patient’s tumor marker levels on September 18, 2021 were as follows: CEA, 2.33 ng/mL, CA-199 3.72 U/mL, and CA-724, 14.11 U/mL. Following a multidisciplinary team discussion–including specialists in surgery, gastroenterology, oncology, and radiology–the patient’s subsequent treatment plan was deliberated. Radiology experts noted a significant reduction in the hepatic flexure tumor compared to previous assessments and recommended a PET-CT scan for further evaluation. Due to concerns about potential tumor-related complications, the surgical team advised the patient to undergo elective surgery to remove the primary lesion. During monotherapy with immunotherapy, the patient experienced no severe immune-related adverse events except for gastrointestinal perforation. The patient concerned about the risks associated with surgery and chemotherapy-related side effects. He chose to continue immunotherapy after thorough discussions with the multidisciplinary team.

**Figure 4:**
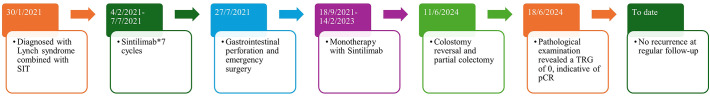
Timeline of the patient’s diagnosis and treatments.

On September 18, 2021, the patient returned to the hospital and continued to receive sintilimab every 21 d until February 2023. Throughout the immunotherapy regimen, the patient underwent regular evaluations, including clinical symptom assessments, laboratory tests, and imaging studies. The results of blood tests are summarized in **Table 1**. CT scans performed in January 2022, April 2022, July 2022, October 2022, September 2023, and April 2024 consistently showed no abnormal enhancement in the colonic hepatic flexure wall. A colonoscopy in April 2024 revealed no tumors in the sigmoid colon and a narrowing of the intestinal lumen at the hepatic flexure. Since colostomies can significantly impact a patient’s daily life, the patient was readmitted on June 11, 2024 for a colostomy reversal. During laparoscopic exploration, the surgical team confirmed narrowing of the colonic hepatic flexure. Consequently, the patient underwent a proximal colectomy and colostomy reversal. Postoperative pathology revealed a scarred area in the colon without residual cancer, with a tumor regression grade (TRG) of 0. The patient is currently recovering well and continues to undergo regular follow-ups and monitoring. The timeline of the patient’s treatment is summarized in **Figure 4**.

**Table 1:** A summary of blood cell counts.

Time	HB (g/L)	PLT (109/L)	PT (s)	D-DIMER (mg/L)	ALT (U/L)	TBIL (μmol/L)	CREA (μmol/L)	CK (U/L)	GLU (mmol/L)	TSH (mIU/L)	CEA (ng/mL)	CA199 (U/mL)	CA724 (U/mL)
2/4/2021	123	455	12.5	0.099	11.8	8.2	83.6	84.7	5.18	1.73	1.97	5.88	29.62
3/4/2021	108	350	11.6	0.088	18.5	5.2	66.2	58.2	5.36	2.01	0.75	5.2	37.46
4/6/2021	103	542	11.7	0.487	12.8	4.1	66.9	50.7	4.86	1.26	0.83	4.03	6.49
4/28/2021	102	288	11.2	0.060	9.9	6.9	70.7	90.3	4.47	1.91	1.48	4.26	5.88
5/21/2021	99	427	10.8	0.188	7.6	3.2	68.3	57.6	4.64	2.05	1.41	3.94	4.87
6/15/2021	100	385	10.4	0.064	12.9	4.6	69.2	127.3	4.69	1.84	2.08	4.44	7.53
7/7/2021	92	361	12.0	0.054	11.4	8.3	80.5	145	4.29	2.41	2.27	5.45	8.08
7/27/2021	106	267	18.2	3.157	15.5	13.4	72.9	N/A	5.87	N/A	2.5	5.33	5.46
9/18/2021	110	344	11.6	0.239	20	6.4	67.8	63.0	4.52	1.46	2.33	3.72	14.11
1/12/2022	123	235	11.1	0.137	11.3	5.9	67.3	67.5	4.73	1.56	2.11	4.78	17.9
4/27/2022	119	261	11.9	0.143	12.6	4.5	71.0	N/A	5.67	1.75	1.97	4.53	10.23
7/21/2022	127	247	12.0	0.100	17.2	5.2	65.9	75.9	4.96	1.90	2.06	5.72	8.74
11/1/2022	129	253	11.5	0.096	15.7	6.4	73.4	61.2	5.21	N/A	1.87	5.98	5.12
8/23/2023	140	241	N/A	N/A	48.6	8.3	79.7	N/A	3.91	N/A	1.82	8.39	3.95

Hb, hemoglobin; PLT, platelet; PT, prothrombin time; ALT, alanine aminotransferase; TBIL, total bilirubin; CREA, creatinine; CK, creatine kinase; GLU, glucose; TSH, Thyroid-stimulating hormone; CEA, carcinoembryonic antigen; CA-199, Carbohydrate antigen 19-9; CA724, Carbohydrate antigen 72-4; N/A, not applicable.

## 3 Discussion

SIT is a rare congenital anomaly characterized by mirror-image transposition of the abdominal and thoracic organs, with an incidence rate of approximately 1 in 8,000 to 1 in 25,000 (4). The exact cause of SIT remains unclear but may be related to hereditary factors, changes in chromosome structure or number, and/or gene mutations. Notably, the absence of Cerberus-like 2 (Cerl2), as well as mutations in DNAAF3 and ZIC3, may have been associated with SIT (10–12). However, the relationship between SIT and tumorigenesis remains unclear. While SIT does not inherently impair organ function, some studies suggest a potential link to cancer through congenital dysfunction of the KIF3 ciliary transport complex, which may disrupt cell signaling pathways (13). However, this hypothesis lacks conclusive evidence and warrants further investigation.

Most patients with SIT have normal organ function and are asymptomatic, with the condition often identified incidentally through imaging studies such as X-rays or CT scans (14, 15). Due to its rarity, physicians often have limited experience in managing these patients. Surgical procedures in SIT cases pose significant challenges, particularly minimally invasive surgeries, due to the altered anatomical orientation of the viscera (16). Advanced imaging modalities such as CT or MRI are crucial for assessing anatomical variations and aiding in surgical planning, especially in emergency situations (17). In this case, the patient required emergency surgery for gastrointestinal perforation, and Laparoscopic exploration revealed transposition of the ascending and descending colons, with perforation of the hepatic flexure. During surgery, the lead surgeon stood on the patient’s right side, while the first assistant stood on the left. Anatomical anomalies often necessitate adjustments in surgical approaches and techniques.

Lynch syndrome is an autosomal dominant hereditary disorder responsible for approximately 3–5% of all CRC, making it the most common hereditary CRC syndrome (2). It results from pathogenic germline mutations in one of four DNA mismatch repair genes (MMR) genes–MLH1, MSH2, MSH6, or PMS2–or from EPCAM deletions. Lynch syndrome predisposes individuals to multiple malignancies, including CRC, endometrial cancer, ovarian cancer, and gastric cancer. Among the MMR gene variants, MLH1 is associated with the highest risk of developing CRC, with a cumulative cancer incidence ranging from 0% (at age 30) to 48.3% (at age 75) in females, and from 4.5% (at age 30) to 57.1% (at age 75) in males (18).

Compared to sporadic CRC, Lynch syndrome-associated CRC presents distinct clinical and histopathological features, including earlier age of onset, faster tumor progression, a higher incidence in the proximal colon, and a greater likelihood of poor histological differentiation, mucinous adenocarcinoma, and signet-ring cell carcinoma (19, 20). Additionally, Lynch syndrome-related tumors often exhibit increased tumor-infiltrating lymphocytes, which are associated with a better prognosis (21, 22). In this case, the patient was diagnosed with bifocal colon cancer at the age of 48. Colonoscopy and pathology confirmed the diagnosis, and genetic testing along with family history analysis led to the identification of Lynch syndrome. To the best of our knowledge, this is the first reported case of bifocal colon cancer associated with both Lynch syndrome and SIT, in which the patient achieved pCR following monotherapy with ICIs.

Due to mismatch repair protein deficiency, DNA mismatches are not corrected efficiently, leading to MSI-H status characterized by base mismatches, sequence insertions, or deletions in microsatellite regions. Tumors can evade immune checkpoints (ICs) expressed on T cells, thereby inducing immune tolerance and inhibiting T-cell proliferation and activation. ICIs function by blocking these ICs, lifting immune suppression of T-cell activation, promoting T-cell proliferation, and ultimately facilitating tumor cell death (23). Research has demonstrated the significant efficacy of ICIs in treating MSI-H/dMMR metastatic CRC (8), prompting exploration of their use in locally advanced MSI-H/dMMR CRC. Multiple prospective studies have reported that neoadjuvant immunotherapy achieves a high clinical and pCR rate (60%–100%) for MSI-H/dMMR CRC, with a relatively low incidence of grade 3 or higher ICI-related toxic reactions (24–26). Given that cancers in patients with Lynch syndrome often exhibit MSI-H status, this finding provides strong theoretical support for the application of ICIs in this population.

The patient received sintilimab treatment for 2 years. 7 months after completing the immunotherapy regimen, follow-up enhanced chest and abdominal CT scans revealed no abnormal enhancement of the colonic hepatic flexure wall, indicating radiological clinical remission. Subsequent routine evaluations showed no abnormal findings, and treatment efficacy was assessed as stable. The surgical specimen revealed no residual cancer, with a TRG of 0, confirming a pCR. This finding further substantiates the efficacy of ICIs in the treatment of Lynch syndrome-associated CRC. This case exemplifies the delayed effects of immunotherapy, a phenomenon frequently observed in clinical trials involving immunotherapeutic agents. In 2013, Chen and Mellman introduced the concept of the “Cancer-Immunity Cycle,” which describes the sequential steps of the anti-cancer immune response. For an effective immune response that leads to tumor destruction, a series of events must be initiated, allowed to progress, and expand iteratively (27). The delayed therapeutic effects of immunotherapy may be associated with T-cell activation, remodeling of the tumor microenvironment, and formation of immune memory (28–30). Delayed clinical effects and long-term survival benefits result in nonproportional hazard models, complicating immunotherapy trial designs, particularly in defining appropriate clinical trial endpoints (31–33). Since responses may be delayed, immune-related adverse events (irAEs) can also manifest later and, in some cases, may not appear during the initial cycles of treatment (34). The delayed effects of immunotherapy represent both a unique advantage and a clinical management challenge.

Although immunotherapies have significantly enhanced patient outcomes across various clinical scenarios, they also impose accompanying risks of toxicity, particularly irAEs. A systematic review indicated that patients undergoing treatment with anti-PD-(L)1 inhibitors develop irAEs at a rate of 74%, with 14% of these classified as grade ≥3 (35). In this case, the patient was monitored for immune-related toxicities during the course of immunotherapy, which included the assessment of clinical symptoms, laboratory tests, and imaging studies. A regular evaluation of the patient’s signs and symptoms, such as fatigue, fever, rash, abdominal pain, and diarrhea, was conducted. Routine laboratory assessments, including complete blood counts, liver function tests, renal function tests, thyroid function tests, coagulation profiles, myocardial enzymes, and blood glucose levels were performed to ascertain whether the function of the corresponding organs was affected. Periodic CT scans were used to evaluate the presence of immune-related conditions, such as pneumonitis and hepatitis. After seven cycles of sintilimab treatment, the patient developed gastrointestinal perforation, which subsequently progressed to a severe infection requiring emergency surgical intervention.

Adverse gastrointestinal reactions are the second most common irAEs. The severity of these events varies widely, ranging from mild to potentially fatal, and includes diarrhea, colitis, ileus, toxic megacolon, and perforation (36–38). Notably, anti-PD-1 or anti-PD-L1 monotherapy was associated with the lowest incidence of significant irAEs, with only 0.9% of patients experiencing severe colitis. Additionally, monotherapy tends to be associated with a reduced recurrence of adverse reactions compared to combination immunotherapy (39, 40). Prompt surgical consultation is essential in patients with severe abdominal pain, toxic megacolon, or bowel perforation. The decision to re-challenge a patient with an ICI should be made in collaboration with a medical oncology team (36).

The duration of immunotherapy must strike a balance between therapeutic efficacy and risk of toxicity. Currently, the optimal timing for treatment cessation remains debatable. In the 5-year follow-up reports of the Keynote-010 and Keynote-024 trials, 83% and 82% of patients, respectively, were still alive 3 years after completing 2 years of pembrolizumab treatment, with 48% and 46% remaining free from disease progression or subsequent therapy (41, 42). The KEYNOTE-177 trial, which focused on colorectal cancer, supports a 2-year cessation strategy, suggesting that some patients maintain progression-free survival after discontinuation of therapy (9). Conversely, the CheckMate-153 trial demonstrated that patients who underwent a period of observation after stopping treatment experienced significantly shorter progression-free survival and overall survival compared than those who continued the therapy (43). Research suggests that for patients who remain progression-free following immunotherapy, it is reasonable to discontinue treatment after 2 years rather than continuing indefinitely (44). Future efforts should focus on optimizing biomarker-guided strategies to balance therapeutic efficacy with safety.

Immunotherapy provided significant therapeutic benefits to this patient; however, the presence of a colostomy led to considerable inconvenience to his daily life, ultimately prompting him to opt for surgical intervention. Studies have indicated that the incidence of severe postoperative complications after neoadjuvant immunotherapy for CRC ranges from 7.8%–21.4% (45–47). In this case, the duration and dosage of immunotherapy administered to the patient exceeded the parameters established in previous studies, suggesting that the rate of adverse events could be higher. To mitigate the risk of surgical complications, the patient underwent 1 year follow-up and monitoring after discontinuing immunotherapy. The interval between immunotherapy and surgery is critical; a duration that is too short may hinder adequate activation of the immune system, while an excessively prolonged interval could lead to tumor progression. Therefore, precise determination of the optimal timing for surgical intervention remains an area that requires further investigation.

In summary, the anatomical variations associated with SIT increase surgical complexity. Advanced imaging modalities, such as CT or MRI, play a crucial role in assessing anatomical structures and facilitating surgical procedures. ICIs represent an effective treatment option for Lynch syndrome-associated CRC, and this report provides further evidence supporting their use. Future studies should explore the optimal timing of immunotherapy, combination treatment strategies, and methods to mitigate immune-related toxicities. These efforts will help establish comprehensive and personalized treatment plans to address the challenges associated with Lynch syndrome management.

## Data Availability

The original contributions presented in the study are included in the article/Supplementary Material. Further inquiries can be directed to the corresponding authors.
